# Intracavitary cisplatin-fibrin followed by irradiation improved tumor control compared to the single treatments in a mesothelioma rat model

**DOI:** 10.1016/j.xjon.2024.07.024

**Published:** 2024-09-04

**Authors:** Michaela B. Kirschner, Mayura Meerang, Vanessa Orlowski, Katarzyna Furrer, Fabienne Tschanz, Ivo Grgic, Virginia Cecconi, Maries van den Broek, Matthias Guckenberger, Martin Pruschy, Olivia Lauk, Isabelle Opitz

**Affiliations:** a Department of Thoracic Surgery, University Hospital Zurich, Zurich, Switzerland; b Department of Radiation Oncology, University Hospital Zurich, Zurich, Switzerland; c Institute of Experimental Immunology, University of Zurich, Zurich, Switzerland

**Keywords:** pleural mesothelioma, intracavitary therapy, chemotherapy, radiotherapy, radiosensitization

## Abstract

**Objective:**

To test the safety and efficacy of combination treatment for pleural mesothelioma (PM) with intracavitary cisplatin-fibrin (cis-fib) plus hemithoracic irradiation (IR) applied after lung-sparing surgery in an orthotopic immunocompetent rat model.

**Methods:**

We randomized male F344 rats into 5 groups: cis-fib (n = 9), 10 Gy IR (n = 6), 20 Gy IR (n = 9), cis-fib+10 Gy IR (n = 6), and cis-fib+20 Gy IR (n = 9). Subpleural tumor implantation was performed on day 0 with 1 million syngeneic rat mesothelioma cells (IL45-luciferase). Tumors were resected on day 9, followed by treatment with intracavitary cis-fib or vehicle control (NaCl-fib). On day 12, computed tomography–guided local irradiation in a single high dose of the former tumor region was applied.

**Results:**

We observed only short-term side effects related to 20 Gy radiotherapy. Compared to 20 Gy, 10 Gy IR did not show an impact on tumor growth. At 3 days after treatment with 20 Gy IR (day 15 of the experiment), we detected significantly smaller tumors in the cis-fib+IR group compared to IR alone (mean tumor growth, 252% vs 539%; *P* = .04). On day 21, there was a significant difference in tumor growth between cis-fib–treated and cis-fib+IR– treated tumors (mean tumor growth, 2295% vs 660%; *P* = .01).

**Conclusions:**

Localized treatment after tumor resection in PM aims to improve local tumor control. Irradiation applied in combination with intracavitary cis-fib in rats is safe up to a dosage of 20 Gy and shows an additive effect on tumor growth delay compared to the single treatments.

## Graphical abstract

**Figure:**
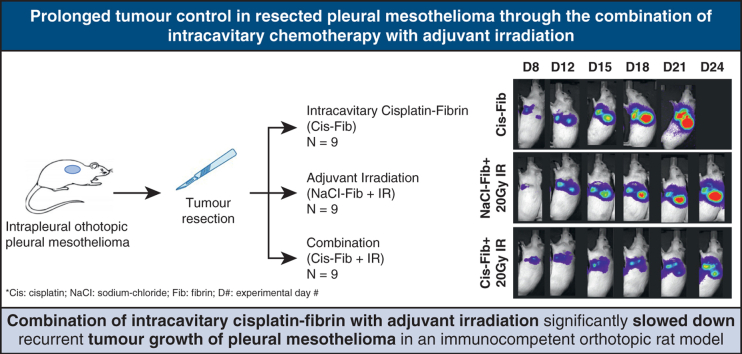

**Figure:**
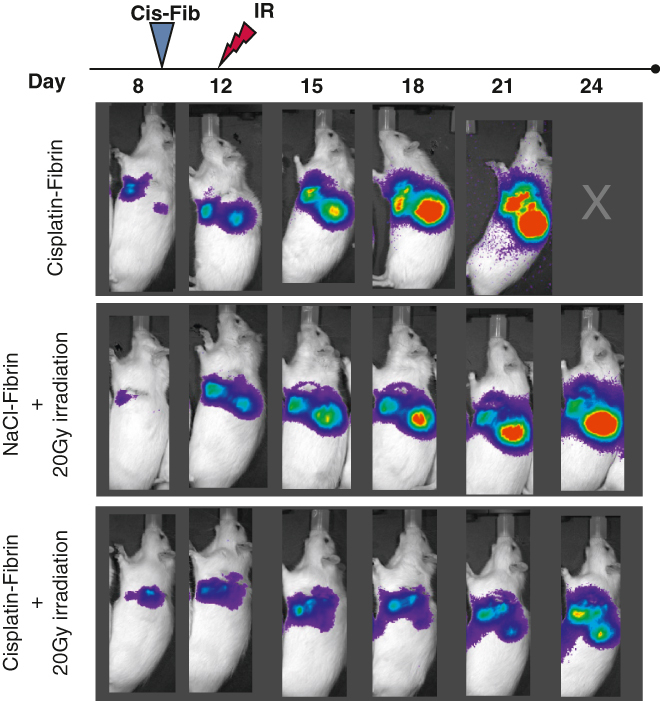
Intracavitary cisplatin-fibrin plus irradiation significantly delayed tumor growth.

**Central Message**

The combination of tumor resection followed by intracavitary chemotherapy and adjuvant irradiation significantly slowed tumor growth in an immunocompetent rat model of pleural mesothelioma.

**Perspective**

Early local tumor recurrence contributes to poor outcomes in pleural mesothelioma. In an orthotopic rat model, combining tumor resection with intracavitary chemotherapy and adjuvant irradiation significantly slowed recurrent tumor growth. With all treatment modalities already tested in clinical practice, this combination holds promise for future clinical use.

Early local tumor recurrence represents a major challenge in the treatment of pleural mesothelioma (PM) and is one of the main reasons for the poor prognosis associated with this devastating pleural cancer.^1^^,^^2^ Patients deemed resectable may be offered a multimodal therapy approach, including macroscopic complete resection. However, anatomic constraints make complete microscopic resection challenging, resulting in a high rate of local tumor recurrence.

Intracavitary treatment combined with surgery potentially could eliminate residual tumor cells, delaying or preventing the onset of local recurrence. The concept of local application of medical treatment has proven very successful in peritoneal cancers, including peritoneal mesothelioma, where chemotherapeutic drugs are being applied, usually in from of hyperthermic intraperitoneal chemoperfusion.^3^ Aiming to decrease treatment-related toxicities that have been seen in the hyperthermic intrathoracic chemotherapy setting in PM,^4^, ^5^, ^6^, ^7^, ^8^, ^9^ we previously developed a novel intracavitary concept. For this, cisplatin is combined with a fibrin gel, which is subsequently sprayed onto the surfaces of the pleural cavity. Following assessment in preclinical animal models,^10^, ^11^, ^12^ we recently confirmed in a phase I clinical trial, INFLuenCe-Meso I,^13^ that this novel concept of intracavitary chemotherapy application is accompanied only by low-level side effects.

Taking into consideration that several studies have suggested a radiosensitizing role of cisplatin,^14^, ^15^, ^16^ most likely through inhibition of the DNA double-strand break repair mechanisms, we sought to evaluate whether this radiosensitizing property could be exploited for the treatment of PM. Using our orthotopic immunocompetent PM rat model previously used to investigate the effect of cisplatin-fibrin on local tumor control following surgical resection, in the present study we investigated the possibility of combining surgical resection followed by intracavitary cisplatin-fibrin with adjuvant irradiation (IR).

## Materials and Methods

### Cell Line and In Vitro Assays

This study used the luciferase-expressing rat mesothelioma cell line IL45-*luc*.^17^^,^^18^ Culture conditions and in vitro assays are described in the Appendix E1.

### In Vivo Animal Experiments

**Figure 1:**
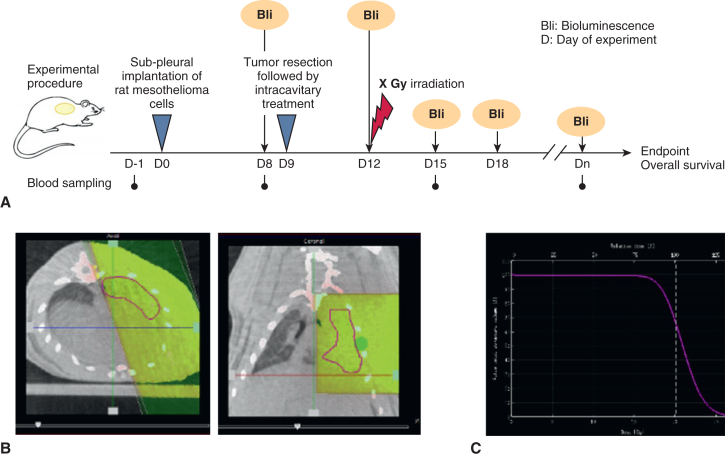
Experimental procedure. A, Scheme for treatment plan and sample collection for experiment with the rat pleural mesothelioma model. B, Example of an irradiation (*IR*) treatment plan for a rat with 20 Gy IR (*red circle*, delineation of lung; *green dot*, target for treatment planning). The area highlighted in *yellow* indicates the IR beam. C, Graph depicting IR deposition in the lung.

The overall scheme for the in vivo experiments is depicted in Figure 1, *A*. All experimental procedures were reviewed and authorized by the Veterinary Office of Zurich, Switzerland (license no. ZH095/2016, approved February 12, 2016) and were performed in accordance with European Union Directive 2010/63/EU for animal experiments. Male Fischer 344 rats (10 weeks old; Charles River Laboratories) were accommodated to the housing facility for at least 2 of weeks acclimatization. On day 0 (D0), 50 μL of freshly prepared sterile cell suspension of 1 × 10^6^ IL45-*luc* cells in Dulbecco’s phosphate-buffered saline (PBS) was implanted underneath the parietal pleura as described previously. IL45-luc generated sarcomatoid tumors in this rat model.^18^ Animals were randomized into 5 groups: cis-fib (n = 9), 10 Gy IR (n = 6), 20 Gy IR (n = 9), cis-fib+10 Gy IR (n = 6), and cis-fib+20 Gy IR (n = 9). Our research question focused on the comparison between 2 treatment modalities (cis-fib and IR); thus, we omitted the untreated control group from this experimental setting. This complies with the 3Rs principle of ethical use of experimental animals, to reduce the number of animals for this research question.

On D9, at which point tumor nodules were visible on bioluminescent imaging in all rats, we performed a lung-sparing tumor resection resembling pleurectomy/decortication in humans. We performed an incision at the injection site and resected the visible tumor nodule while avoiding damage to the lung. Cisplatin-fibrin was prepared as described previously^10^^,^^12^ and applied immediately on the resected area on completion of the resection.^10^^,^^12^ Animals in the IR groups underwent irradiation at 3 days after the operation (D12).

### Local Intracavitary Treatment

For each rat, 0.2 mL of the cisplatin-fibrin gel (containing 0.1 mg of cisplatin) was applied to cover the entire resection site (∼2 cm^2^). Details on the preparation of cisplatin-fibrin are provided in the Appendix E1. This local dose of 0.05 mg/cm^2^ of resected pleural surface area is equivalent to the dose previously tested in our pig model (5 mg/100 cm^2^ pleural surface) that showed no toxicity^11^ and corresponds to approximately 33 mg/m^2^ of body surface area tested in our phase I clinical trial.^13^

### Image-Guided Local IR

A single dose of IR was delivered at a rate of 3 Gy/min with an image-guided stereotactic small animal irradiation unit (Precision X-Ray, X-Rad SmART 225kV unit, equipped with a cone beam CT scanner), as described in the Appendix E1.

### In Vivo Imaging and Signal Quantification

The monitoring of tumor burden by bioluminescent imaging (Bli) was performed on D8 (before tumor resection), on D12, and every 3 days thereafter until the termination of the experiment. Because only tumor cells were transfected with vectors expressing luciferase, the bioluminescent signal reflects numbers of tumor cell proportionally^19^ (Appendix E1).

### Health Monitoring and Assessment of OS

After surgery, animal health and well-being were monitored daily, blinded for altered health condition. In brief, animals were monitored for signs of side effects resulting from chemotherapy and radiotherapy, including changes in body weight, body condition score, sign of distress and pain, and signs of respiratory problems, such as blue/white extremities due to desaturation or increased breathing rate. Overall survival (OS) was defined as the time from tumor implantation until the end of the experiment when at least 1 of the termination criteria was met (Table E1).

### Histologic Analysis of Tumor and Lungs

We harvested tumors and the lungs and preserved by formalin fixation. Histology of the lungs were assessed by hematoxylin and eosin staining. The tumor proliferation rate was determined using Ki-67 staining on paraffin-embedded sections (Appendix E1).

### Analysis of Blood Cell Components

Sublingual blood was collected on the day before tumor implantation (D-1), the day before tumor resection (D8), at 3 days after irradiation (D15), and prior to euthanasia for analysis of overall leukocyte, neutrophil, and lymphocyte counts. Details of the FACS analysis are provided in the Appendix E1.

## Results

### Additive and Radiosensitization Effects of Cisplatin In Vitro

**Figure 2:**
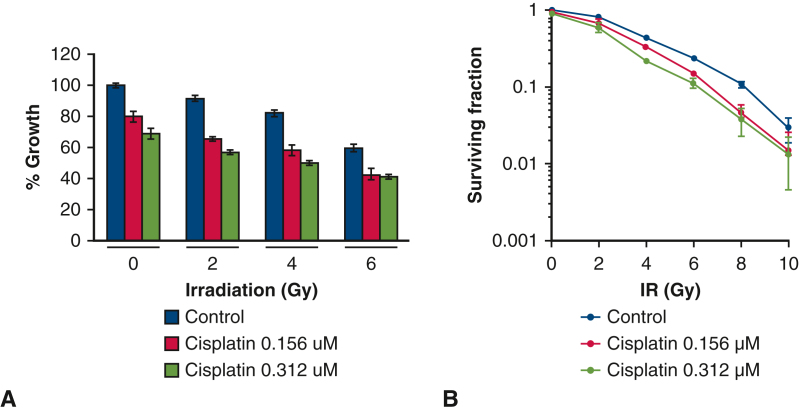
Additive effect of cisplatin (*cis*) and irradiation (*IR*) in vitro. A, Dose response of IL45-*luc* cells to combined treatment of cis plus IR based on viability methylthiazolyldiphenyl-tetrazolium bromide assay after 72 hours of treatment. B, A colony-formation assay performed at day 7 after treatment with increasing dosages of IR alone or in combination with cis. Percent cell growth and surviving fraction of each treatment were normalized to the untreated control (no cisplatin and IR treatment). The data are plotted as mean ± SD from at least 2 independent experiments.

We first evaluated the efficacy of the combined cisplatin and IR treatment in IL45-*luc* in vitro. We observed a dose-dependent response to the combined treatment and detected an additive effect after short-term treatment (72 hours) as assessed by a cell viability assay (Figure 2, *A*). A colony-formation assay was performed to identify the effect of single cell proliferation for longer treatment (7 days) with the cisplatin–IR combination (Figure 2, *B*). We calculated the combination index (CI) using the data generated from CFA using CompuSyn software and detected a synergistic effect (CI < 1) of 0.155 μM cisplatin and IR starting at 8 Gy (CI = 0.95) and more profoundly at 10 Gy (CI = 0.62). These data suggest a radiosensitising effect of cisplatin in IL45-*luc* cells when combined with high-dose radiation.

### Safety of Combined cis-fib+IR in Rat PM Model

**Figure 3:**
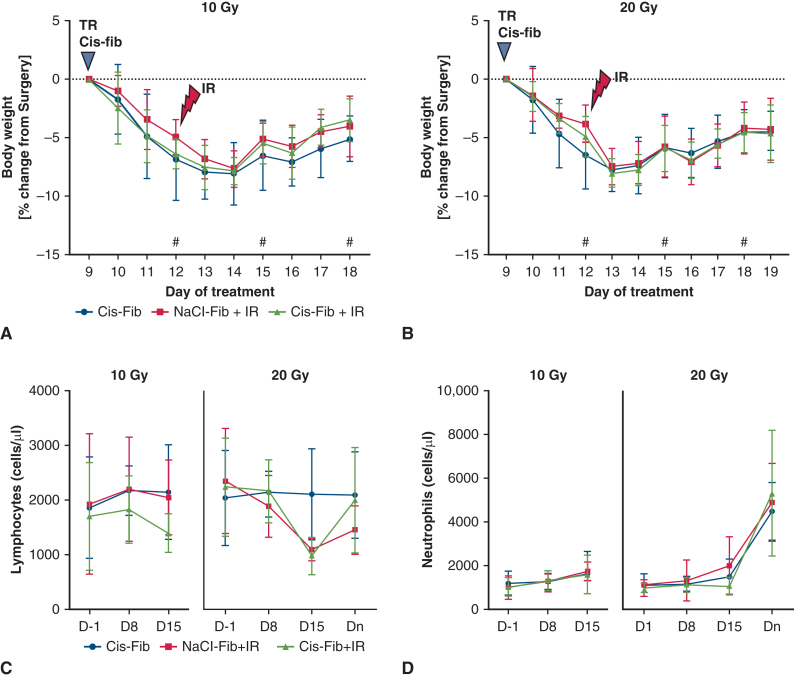
Effects of treatments on body weight and white blood cell count. A and B, Percent daily body weight change compared to body weight before tumor resection surgery and local treatment with cisplatin-fibrin (*cis-fib*) (D9). C and D, Absolute lymphocyte counts and absolute neutrophil counts measured on D-1 (before tumor implantation), D8 (before tumor resection), D15 (3 days post-IR), and Dn (prior to euthanasia). The data are displayed as mean ± SD. Data on Dn are available only for the cis-fib, 20 Gy IR, and cis-fib+20 Gy IR groups. *TR*, Tumor resection; *IR*, irradiation. #Bli imaging.

All animals were monitored closely and daily after treatment for signs of side effects resulting from chemotherapy and radiotherapy (eg, changes in body weight, body condition score, signs of distress, pain) and for signs of reduced pulmonary function. We found a loss of body weight in all animals immediately following tumor resection surgery, which continued until D14. There was no difference in weight loss between the cis-fib and cis-NaCl groups. None of the animals showed deterioration of body conditioning or activity score in the immediate postinterventional phase or any signs of pulmonary side effects. On D12, animals were subjected to Bli measurement under general anesthesia, along with IR that caused further weight loss (Figure 3, *A* and *B*). Treatment with a single dose of 20 Gy or cis-fib+20 Gy IR caused significantly more weight loss on the day after treatment (D13 vs D12) compared to the no IR group, but all animals regained this weight within 2 days (Figure 3, *B*).

**Figure E1:**
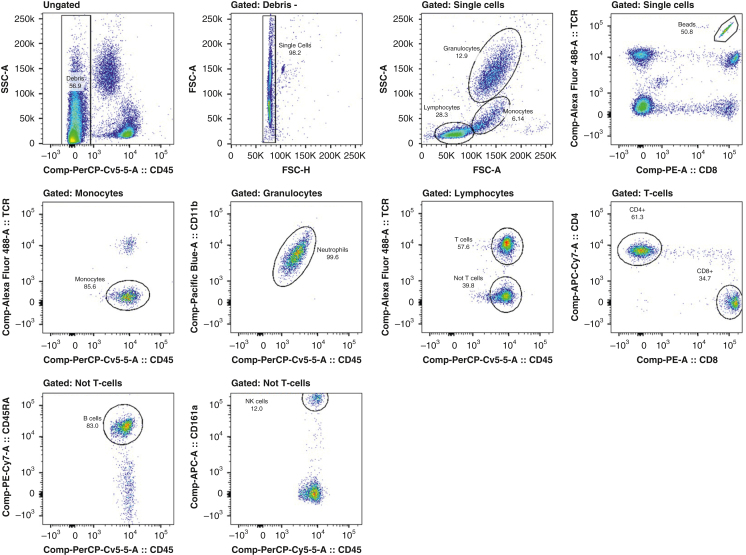
Flow cytometry analysis of blood cell component.

White blood cell count revealed a drop in lymphocyte count at D15 (3 days after IR) following 20 Gy IR but not after 10 Gy IR or cis-fib alone (Figure 3, *C*). Combination treatment with cis-fib followed by 20 Gy IR caused a drop in lymphocyte count to the same extent as 20 Gy IR alone (Figure 3, *C*). The decrease also applied to all lymphocyte subpopulations (CD4^+^, CD8^+^, and natural killer [NK] cells) (Figure E1). Lymphocyte counts were again increased to levels comparable to those before IR at the time of euthanasia. Neutrophil counts did not change following both 10 Gy IR and 20 Gy IR (Figure 3, *D*). Neutrophil counts were significantly elevated at the time of euthanasia, indicating an ongoing inflammatory process resulting from tumor growth.

**Figure E2:**
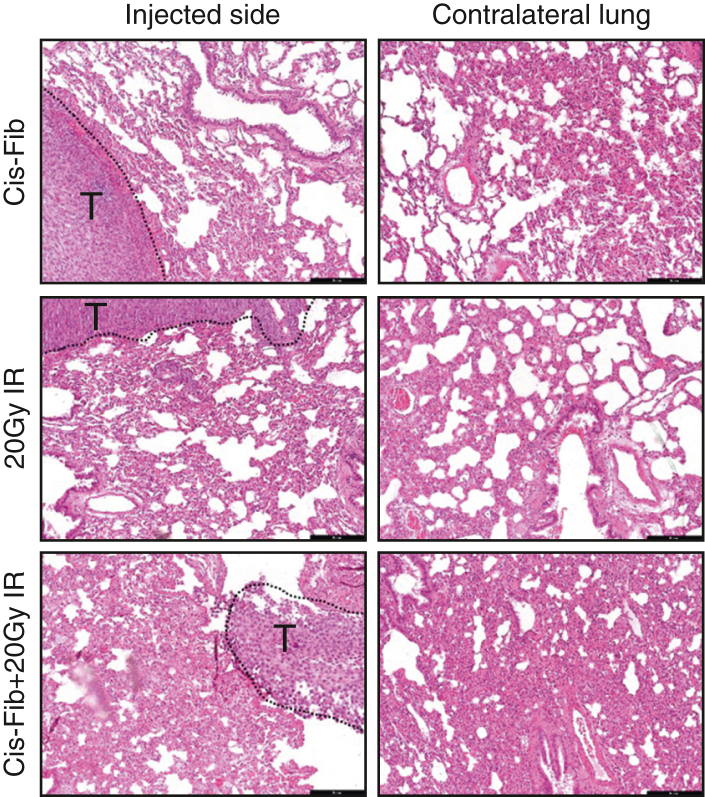
Hematoxylin & eosin staining of the lungs collected at the endpoint of the experiment. The side implanted with IL45-*luc* cells (injected side) showed a tumor mass (T) in the lung that was inflamed due to tumor invasion and collapsed alveoli in all groups. At the endpoint, tumors also invaded into the contralateral pleural space and induced inflammatory reaction in the contralateral lungs. There was no increased fibrosis in the irradiated groups compared to the nonirradiated group. *cis-fib*, Cisplatin-fibrin; *IR*, irradiation

We further analyzed the effect of IR on the underlying lungs assessed at the endpoint and found no increased fibrosis in the irradiated groups compared to the nonirradiated group (Figure E2 and Appendix E1, Results).

### Treatment Efficacy

**Figure 4:**
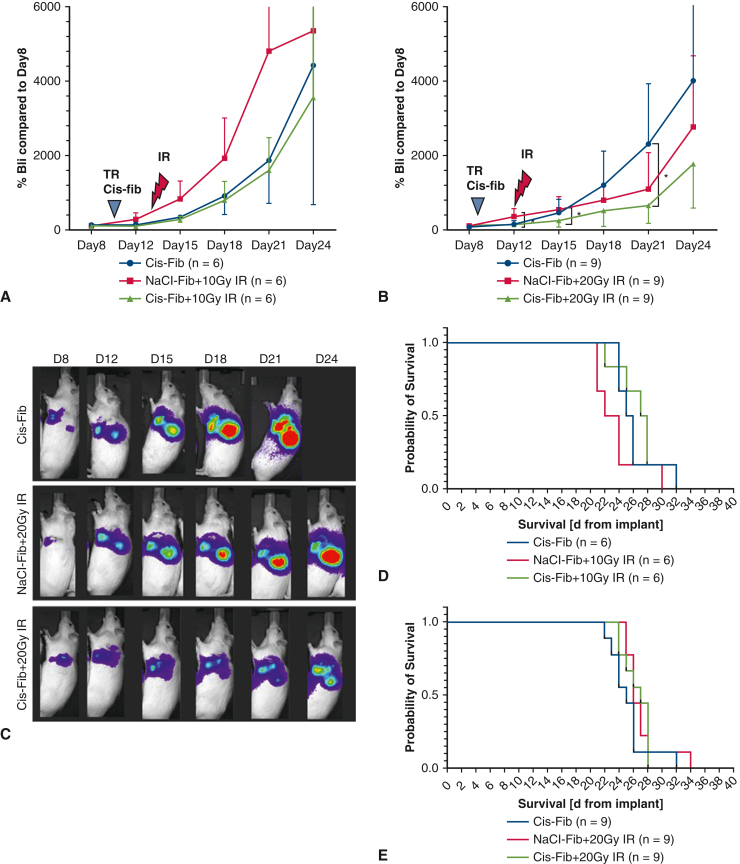
Additive effect of cisplatin (*cis*) and irradiation (*IR*) on tumor control in the PM rat model. A, Tumor burden assessed by bioluminescent (*Bli*) imaging at 3 days after treatment with local cis-fib followed by 10 Gy IR. The plot represents percentage of tumor burden (% Bli) compared to tumor burden before tumor resection (D8). No additive effect is detected at this dose level. B, Additive effect of cisplatin and IR detected when 20 Gy IR was applied after cis-fib treatment (see the differences in growth rate starting at D15). C, Representative images of Bli. D, The animal in the cis-fib group reached the termination endpoint prior to D24. E, Overall survival of animals plotted from the day of implantation. The data are presented as mean ± SD. *TR*, Tumor resection; *cis-fib*, cisplatin-fibrin; *IR*, irradiation. ∗*P* < .05; unmarked, not statistically significant by unpaired *t* test.

To evaluate treatment efficacy, we assessed 2 different endpoints: tumor growth, monitored by repeated Bli imaging every 3 days, and OS. We implanted tumor cell suspension underneath the subparietal pleura to create a single tumor. On day 8 after implantation, in the majority of animals, Bil imaging detected a single large nodule at the injection site, along with smaller detectable nodules in some animals (Figure 4, *C*). Cis-fib alone caused a delay in tumor growth up until D15 (Figure 4, *A*, compare growth curve from D8 to D12 and D15). We started with a low IR dosage (10 Gy) and detected comparable tumor growth in animals treated with 10 Gy IR compared to no IR (the cis-fib group) (Figure 4, *A*; compare D12 vs D15). Accordingly, there was no significant improvement in OS with cis-fib+10 Gy IR compared to the single treatments (Figure 4, *D*). Thus, we decided to escalate to 20 Gy IR after treating 6 animals per group.

At 3 days after treatment with 20 Gy IR (D15), we detected significantly smaller tumors in the cis-fib+IR group compared to the IR-alone group (mean tumor growth, 252% vs 539%; *P* = .04). On D21, there was a significant difference in tumor growth between cis-fib–treated and cis-fib+20 Gy IR–treated tumors (mean tumor growth, 2295% vs 660%; *P* = .01) (Figure 4, *B*). Accordingly, the greater delay in tumor growth of IR-treated tumors was reflected in a flattening of the growth curve up until D21. This growth delay effect of IR diminished after D21 (9 days post-IR). Representative Bli images are shown in Figure 4, *C*. Although significant improvement in tumor growth control was achieved, we detected no difference in OS with cis-fib+20 Gy IR compared to cis-fib alone or to 20 Gy IR alone (Figure 4, *E*).

## Discussion

**Figure 5:**
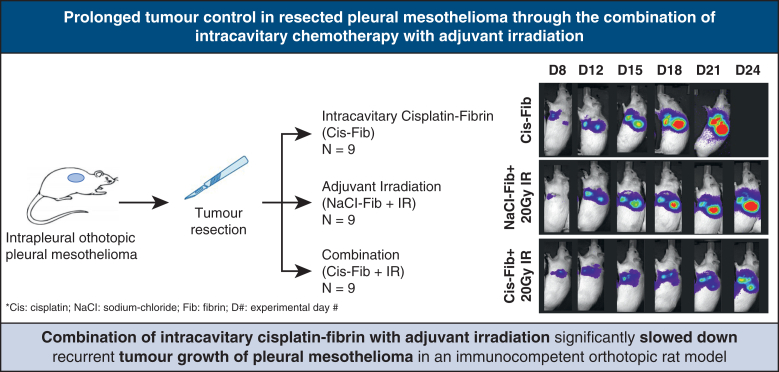
Intracavitary chemotherapy (cis-fib) plus adjuvant irradiation (20 Gy IR) significantly delays tumour growth compared to single treatment groups.

The present study demonstrates that the combination of cis-fib followed by 20 Gy IR is safe and resulted in a significant tumor growth delay compared to the single treatments in our orthotopic rat model of PM (Figure 5). Owing to the widespread and diffuse growth of the tumor along the pleura, achieving microscopic complete resection is almost impossible in PM. Thus, additional treatment approaches, in form of, for example, providing local treatment as part of surgical resection and targeting the remaining microscopic tumor, are urgently needed to reduce early recurrent outgrowth.

The feasibility and efficacy of local irradiation have been investigated in several clinical trials for PM^20^, ^21^, ^22^, ^23^; however, due to the sensitivity of the underlying organs and high risk of irradiation-related toxicities, not every patient is eligible for this adjuvant therapy. Determination of appropriate timing before and after surgery has been evaluated in several clinical trials, but the results are insufficient to allow for a clear conclusion.^23^, ^24^, ^25^ Significantly improved survival outcomes of radiotherapy provided before surgery was shown in the SMART clinical trial.^26^ For radiotherapy given postoperatively, the SAKK 17/04 trial investigated fractionated radiotherapy following surgery (extrapleural pneumonectomy) versus postoperative observation and showed that radiotherapy was associated with a marginal improvement in median locoregional relapse-free survival (9.4 vs 7.6 months).^27^

Initial in vitro experiments on rat IL-45 PM cells confirmed findings for other cancers^16^^,^^28^ that pretreatment with cisplatin can sensitize to IR. Using an orthotopic rat model of PM, we further aimed to identify whether adding local cisplatin could improve the effect of IR administered after surgery.

The data from this animal study show that adding the 2 local treatment modalities after surgical tumor resection is not associated with an increased risk of treatment-related toxicities. IR-related toxicities (eg, decreased body weight and lymphocyte count) were observed after 20 Gy, but these effects were only transient. Because the rats receiving 10 Gy IR alone showed stronger tumor growth compared to those treated with cis-fib alone (without IR), we conclude that this IR dosage is not sufficient to achieve local tumor control. Application of 20 Gy adjuvant IR resulted in slightly better local tumor control than cis-fib alone. Thus, our data suggest an additive effect of the 2 local treatment modalities. Although these data certainly require validation, we have shown that it is possible to increase the local tumor control achieved by adding adjuvant IR after cis-fib.

Although the data from our Bli analysis^18^ showed a significant additive effect of intracavitary cis-fib and 20 Gy IR, resulting in significantly delayed tumor growth compared to the single treatments, we did not observe a difference in OS between the treatment groups. The underlying reasons for this lack of translation into survival are most likely attributable to the intense schedule of our animal experiments. Stress caused by anesthesia and resulting weight loss likely contributed to the overall well-being of all animals independent of their tumor burden or treatment schedule; therefore, OS cannot be attributed specifically to the tumor burden.

### Limitations

The setup of the rat model requires further adjustments. In human patients, IR is usually given in smaller fractions, not as single high dose. Hypofractionated irradiation might result in different outcomes. The aim of this model was to create a similar scenario for local recurrence, the original problem of treatment for PM. The generation of single tumor nodules allows us to better control and monitor the recurrence after resection and treatment. Nevertheless, some leakages resulting in smaller nodules that were not macroscopically visible at resection might result in a high standard deviation of tumor growth observed. In this experimental setting, analysis of tissues was possible only at the conclusion of the experiment, the stage at which tumor regrowth has already occurred. Thus, the analysis of tissues at this late time point did not provide additional informative data on response to treatment on cellular and molecular levels.

## Conclusions

Cis-fib has been deemed feasible and safe in phase I and II clinical trials at our institution. Here we further demonstrate that adjuvant radiotherapy provided after local intracavitary treatment with cis-fib is safe and suggest a benefit of this combined treatment for tumor control in this rat recurrence model.

## Conflict of Interest Statement

I.O. reports receiving institutional grants from Roche, Medtronic, and XVIVO; serving on a steering committee for Roche Genentech and on advisory boards for AstraZeneca, MSD, BMS, Medtronic, and Regeneron; serving as a proctor for Intuitive; and receiving speaking fees from Intuitive, Sanofi, and Siemens. All other authors reported no conflicts of interest.

The *Journal* policy requires editors and reviewers to disclose conflicts of interest and to decline handling or reviewing manuscripts for which they may have a conflict of interest. The editors and reviewers of this article have no conflicts of interest.

## Figures and Tables

**Table E1:** Termination criteria

Discontinuation criteria
1. Body conditioning score 2^E3^ 2. Weight loss ≥15% from tumor resection 3. Activity score 3, “no activity even upon stimulation” 4. The presence of blue or white colored ear/extremities/eyes (lack of oxygen) 5. The presence of stress and pain and not relieved within 24 h after administration of pain medication 6. Severe breathing problem score 3 7. Severe diarrhea that persists for more than 24 h

**Table E2:** Antibodies for blood cell analysis by flow cytometry

Antibody	Supplier	Catalog no.	Dilution	Channel	Marker for
CD45 PerCP/Cy5.5	BioLegend	202220	1:100	PerCP/cy5.5	Leukocytes
CD11b BV450	BD	562108	1:100	Pacific Blue	Granulocytes
CD161a APC	BioLegend	205606	1:100	APC	NK cells
TCR α/β FITC	BioLegend	201105	1:100	Alexa 488	T cells
CD4 APC/Cy7	BioLegend	201517	1:100	APC/Cy7	CD4^+^ T cells
CD8a PE	BioLegend	200607	1:100	PE	CD8^+^ T cells
CD45RA PE/Cy7	BioLegend	202315	1:100	PE/Cy7	B cells

## Appendix E1 Materials and Methods

### Cell Line and In Vitro Assays

The rat mesothelioma cell line expressing luciferase (IL45-*luc*) was generated by stable transfection of the IL-45 cell line, originally cultivated from peritoneal mesothelioma tumors of an F344 rat exposed to crocidolite.^E1^^,^^E2^ The tumors generated by IL-45-*luc* were comparable to those of IL-45.^E2^ Cells were maintained in RPMI 1640 medium (Biowest; L0498) containing 1 mM sodium pyruvate (Biowest; L0642-100), penicillin/streptomycin (Biowest; L0022), 200 μg/mL Geneticin (Gibco; G418 Sulfate 11811-023), and 10% fetal bovine serum at 37 °C with 5% CO_2_. Cells were tested to be free of mycoplasma and rodent pathogens. For measurement of cell survival, at 24 hours after seeding in 96-well plates (3000 cells/well), cells were exposed to 0.156 μM and 0.312 μM cisplatin in NaCl solution (Actavis) in complete medium for 2 hours, followed by exposure to increasing doses of irradiation ranging from 2 Gy to 10 Gy (Xstrahl 200 kV X-ray unit) at 1 Gy/min at room temperature. Measurement of cell survival by methylthiazolyldiphenyl-tetrazolium bromide (MTT) assay was performed after additional 72 hours of incubation at 37 °C in 5% CO_2_. For the colony-formation assay, cells were seeded in 6-well plates for 24 hours (seeding cell doses: 150 cells for 0 Gy and 2 Gy, 600 cells for 4 Gy and 6 Gy, 1000 cells for 8 Gy, and 1500 cells for 10 Gy) and were subjected to treatment with cis followed by IR using the same procedure described above. After treatment, cells were incubated for an additional 7 days at 37 °C in 5% CO_2_. Finally, cells were fixed and stained with crystal violet and counted manually for visible colonies. The surviving fraction (%) was calculated by comparing plating efficiency (number of cells plated/number of colonies) to that in untreated non-IR controls.

### Surgery

All surgical procedures were performed using isoflurane for both induction and maintenance of anesthesia. Following induction anesthesia, rats were intubated with an 18-gauge polyethylene angiocatheter connected to a small animal ventilator (model 683; Harvard Apparatus), and ventilated using a tidal volume of 10 mL/kg, a respiratory rate of 75/minute, −3 cm H_2_O positive end-expiratory pressure, and 2 to 3 vol% isoflurane in 750 mL/min O_2_. In addition, local anesthesia of 1:1 lidocaine plus bupivacaine (6 mg/kg lidocaine and 3 mg/kg bupivacaine) was applied. For systemic analgesia during tumor implantation, rats received paracetamol (30 mg/kg) at 30 to 60 minutes before and at 4 hours after the operation. For tumor resection, rats received paracetamol before and buprenorphine (Temgesic, 0.05 mg/kg) immediately after the operation, followed by burprenorphine every 6 hours and in drinking water at night (0.05 mg/mL buprenorphine in drinking water) until D11. In addition, rats received antibiotics in drinking water (400/80 mg Bactrim diluted in drinking water) for 5 consecutive das after the operation. We expected a final dose of 53 mg/kg/24 hours (http://www.bu.edu/researchsupport/compliance/animal-care/working-with-animals/additives-to-the-drinking-water-for-rats-and-mice-iacuc/); for prevention of possible infection from a weakened immune system resulting from chemotherapy or radiotherapy. Details of the discontinuation criteria are provided in Table E1.

### Local Intracavitary Treatment

Fibrin was prepared from sterile human fresh frozen plasma blood group 0 using a Vivostat fibrin processing kit and PRO-800 processor (Vivostat SA) and stored at −20 °C until use. For intracavitary administration of cis-fib, 1 syringe containing cisplatin (Actavis, 1 mg/mL in 0.9% NaCl) or NaCl mixed with fibrin (1:1) and 1 syringe containing buffer (pH 10) were loaded to the Vivostat application system. Using this system, the 2 components were sprayed to the resected tumor area and the intrathoracic cavity, covering the lung. A change in pH achieved through mixing of the components immediately before leaving the spray tip induced clotting of fibrin and formation of cis-fib gel or NaCl-fib on the target surface area.

### Image-Guided Local IR

Based on a CT scan, an individual treatment plan was designed with the corresponding SmART Plan software to achieve the highest dose accuracy at the resection site while sparing vital organs, including heart and lungs (Figure 1, *B* and *C*). Radiotherapy was applied with 2 opposing fields using a 25 × 30-mm collimator. Animals were under isoflurane anesthesia during the entire session, including planning and irradiation, which took approximately 20 minutes per animal and was performed by staff trained in use of the X-Rad SmART system.

### In Vivo Imaging and Signal Quantification

Prior to the imaging session, rats were injected intraperitoneally with 150 mg/kg of sterile filtered Xenolight d-Luciferin –K^+^ salt bioluminescent substrate (PerkinElmer; 122796) dissolved in Dulbecco’s PBS. Bli measurement was recorded starting 15 minutes after the injection using an IVIS Spectrum equipped with Living Image v 4.7.1 software (PerkinElmer). Optimal exposure time was adjusted automatically by the software to prevent underexposure or overexposure. When the maximum Bli signal was reached, the region of the tumor was marked and measured for Bli signal intensity (total flux; photons/second).

### Analysis of Blood Cell Components

Sublingual blood was collected in K3 EDTA tubes with capillary caps on the day before tumor implantation (D-1), the day before tumor resection (D8), 3 days after IR (D15), and prior to euthanasia for analysis of overall leukocyte counts, as well as neutrophil (granulocytes) and lymphocyte (NK cells, B cells, T cells) counts. Blood was stored at 4 °C until analysis within 72 hours after collection. For analysis, 20 μL of whole blood was resuspended in 2 mL of PBS. After centrifugation at 400 × *g* for 5 min, cell pellets were resuspended in 50 μL of PBS and incubated with the antibodies listed in Table E2 for at least 30 minutes at room temperature or stored for up to 24 hours at 4 °C. Afterwards 1-stepFix/Lyse solution (eBioscience) was added for at least 30 minutes to up to 48 hours at 4 °C. Samples were then washed in 2 mL of PBS and finally resuspended in 400 μL of PBS. Then 100 μL of 123count eBeads (Invitrogen) were added, and samples were filtered through 20-μm sieves prior to flow cytometry analysis (FACS Canto II; BD Biosciences). We used compensation beads prepared according to the manufacturer’s instructions (UltraComp eBeads; Affymetrix). Data were analyzed with FlowJo v10.6.1 (BD Biosciences). Figure E1 shows gating and selection of cell populations. A CD45+ (leukocytes) single cell population was selected for gating. We analyzed lymphocyte subpopulations including T lymphocytes (TCR α/β^+^; CD4 T cells and CD8 T cells), CD45RA (B cells), and CD161a (NK cells). Neutrophils were gated by size and granularity followed by CD11b staining. Beads were identified and counted in fluorescein isothiocyanate (FITC) and phycoerythrin (PE) channels. Absolute blood cell count (cells/μL) was calculated using the following equation:

$$absolute\;cell\;count=\frac{cell\;count*eBeads\;volume}{eBead\;count*blood\;volume}*eBeads\;concentration.$$

### Immunohistochemical Staining

Formalin-fixed, paraffin-embedded sections (3 μm) were deparaffinized and rehydrated. Antigen retrieval was achieved using target antigen retrieval pH 9 solution (Dako; K8005) and stained with anti-Ki-67 (Abcam; 16667, 1:200 dilution), followed by Dako EnVision rabbit/HRP/DAB system and counterstained with hematoxylin. The staining was visualized with a Leica AF 6000B light microscope, and 5 random images were captured. Ki-67^+^ cells were counted manually in all 5 images using ImageJ 1.52a in a blinded fashion.

## Results

### Assessment of Lung Histology

We collected the lungs at euthanasia to assess for any signs of adverse effects resulting from chemotherapy or radiotherapy, including pneumonitis or lung fibrosis. There were no differences in macroscopic signs of increased pneumonitis or fibrosis between treated and untreated sides or between single-agent and combination treatments (Figure E2). We also performed a preliminary analysis of cell proliferation with Ki-67 staining of the tumor tissues collected at the earliest OS of the 3 groups (D24 and D25). From analysis of 2 rats per group, mean Ki-67 staining was 58 ± 11.4% for cis-fib, 74 ± 5.4% for 20 Gy IR alone, and 70 ± 14.2% for cis-fib+20 Gy IR. Because we did not observe any difference in this preliminary analysis, and because the data also corresponded to the Bli results that showed no difference in growth rate after D21 (Figure 4, *B*), we did not perform this analysis on the remaining samples.
